# Complete Genomic Sequence for an Avian Group G Rotavirus from South Africa

**DOI:** 10.1128/genomeA.00107-15

**Published:** 2015-03-12

**Authors:** Karla M. Stucker, Timothy B. Stockwell, Martin M. Nyaga, Rebecca A. Halpin, Nadia Fedorova, Asmik Akopov, Harry Ngoveni, Ina Peenze, Mapaseka L. Seheri, M. Jeffrey Mphahlele, David E. Wentworth

**Affiliations:** a J. Craig Venter Institute, Rockville, Maryland, USA; b South African Medical Research Council, Diarrhoeal Pathogens Research Unit (MRC-DPRU), Faculty of Health Sciences, Sefako Makgatho Health Sciences University, MEDUNSA, Pretoria, South Africa

## Abstract

We report the first complete sequence for an avian group G rotavirus (RVG) genome from Africa, which is the third publically available RVG genome. These RVG genomes are highly diverse, especially in their VP4, VP7, NSP4, and NSP3 segments, indicating that RVG diversity is comparable to that of rotavirus A.

## GENOME ANNOUNCEMENT

Rotaviruses cause gastrointestinal diseases in many vertebrate species worldwide. As members of the *Reoviridae* family, rotaviruses have double-stranded RNA (dsRNA) genomes that comprise 11 segments. Eight rotavirus groups/species—rotaviruses A through H—have been defined by their VP6 proteins (1). Here, we report the first complete avian group G rotavirus (RVG) genome from Africa.

The RVG genome was extracted and amplified from a semi-diarrheal fecal sample collected from an otherwise asymptomatic 5-month-old female chicken (Gallus gallus domesticus) in Nkomo Village, Giyani, South Africa in 2011 using a sequence-independent amplification technique (2). Briefly, nucleic acids were extracted using TRI Reagent LS, and the dsRNA genome was enriched using lithium chloride precipitation. The PC3-T7 loop adapter was ligated to the 3′ ends of the dsRNA segments using T4 RNA ligase, and the cDNA was PCR amplified using primer PC2 (2). The amplified genome segments underwent standard bar coding and library construction for sequencing on the Ion Torrent, as well as a separate sequence-independent single-primer amplification (SISPA) using bar-coded random hexamers (3, 4) for sequencing on Illumina MiSeq v2 and HiSeq 2000.

Sequencing reads from all platforms were sorted by bar code, and bar codes and random hexamers were trimmed prior to assembly. Reads from all sequencing platforms were combined and *de novo* assembled using CLC Bio’s clc_novo_assemble program. Assemblies were performed before any RVG genomes were publically available; therefore, distant homology searches using tblastx and HMM methods were used to assign contigs to the correct rotavirus segments. Once initial *de novo* contigs were identified for each segment, iterative cycles of mapping assembly for each segment allowed them to be built out through their termini using CLC Bio’s clc_ref_assemble_long program to identify additional reads and extend the termini of each segment, followed by *de novo* assembly using all mapped reads. The iterations halted when each of the segment contigs reached the canonical GG at the 5′ terminus and CC at the 3′ terminus (5), with the PC2 primer sequence immediately beyond the termini. For this genome, all segments shared a 9-bp conserved 3′ terminal sequence (5′-TAAAGACCC-3′). For two regions each in VP1 and VP2, primers were designed and Sanger sequencing performed to improve coverage and connect contigs. The final assemblies were annotated using VIGOR (6, 7) and submitted to GenBank.

Comparing this African RVG genome with the recently deposited GenBank reference RVG genome from Germany (accessions NC_021580 through NC_021590) (8) shows a high degree of sequence diversity, with nucleotide percent identities ranging from 55.58% for VP4 to 94.54% for NSP5 and amino acid percent identities ranging from 40.73% for VP4 to 98.21% for VP6. As is typical for rotavirus species, the VP4, VP7, NSP4, and NSP3 segments are the most diverse among the 3 available RVG genomes from South Africa, Germany, and Hong Kong (8, 9). These comparisons demonstrate that a large amount of RVG diversity exists in nature and much of it remains to be sequenced.

### Nucleotide sequence accession numbers.

The genomic sequence data are deposited in GenBank under the accession numbers KJ752079 through KJ752089.
